# The use of implementation strategies to promote the uptake of psychological therapies among people with multiple sclerosis: A scoping review protocol

**DOI:** 10.1371/journal.pone.0337105

**Published:** 2025-11-20

**Authors:** Ashvene Sureshkumar, Robert Simpson, Mark Bayley, Monika Kastner, Jillian Scandiffio, Emilia Main, Claire Zhang, Harzaan Gnanakaran, Joshua Wijeratne, Alesha Saxena, Sarah Munce

**Affiliations:** 1 Rehabilitation Sciences Institute, University of Toronto, Toronto, Ontario, Canada; 2 KITE Research Institute, Toronto Rehabilitation Institute - University Health Network, Toronto, Ontario, Canada; 3 North York General Hospital, Toronto, Ontario, Canada; 4 Institute of Health Policy, Management and Evaluation, University of Toronto, Toronto, Ontario, Canada; 5 Temerty Faculty of Medicine, University of Toronto, Toronto, Ontario, Canada; 6 McMaster University, Hamilton, Ontario, Canada; 7 University of Toronto, Toronto, Ontario, Canada; 8 Bloorview Research Institute, Holland Bloorview Kids Rehabilitation Hospital, Toronto, Ontario, Canada; Cleveland Clinic, UNITED STATES OF AMERICA

## Abstract

**Objective:**

This paper aims to standardize a scoping review protocol for completion of a scoping review. The proposed scoping review aims to: 1) determine the extent of the literature on which implementation strategies have been used to develop, deliver, and sustain psychological interventions for people with multiple sclerosis (PwMS) and 2) investigate how equity, diversity, inclusion and accessibility considerations are embedded within these implementation strategies and psychological interventions more broadly.

**Introduction:**

People with multiple sclerosis experience high levels of stress, anxiety and depression. Psychological interventions, such as mindfulness-based interventions have been shown to be effective in managing these symptoms, yet their implementation in clinical practice is underexplored. Investigating the implementation of psychological interventions can help contextualize the efficacy and impact of these programs for PwMS. This proposed review aims to fill this knowledge gap by determining which implementation strategies have been used to develop and deliver psychological interventions for PwMS.

**Methods:**

This scoping review will follow the Joanna Briggs Institute (JBI) methodology and reporting will follow the Preferred Reporting Items for Systematic Reviews and Meta-analyses extension for scoping review (PRISMA-ScR). The search will be conducted across MEDLINE, Embase, Cumulative Index to Nursing and Allied Health Literature (CINAHL), and PsychInfo (Scopus). Two reviewers will independently conduct screening and data extraction in duplicate, with any disagreements resolved through discussion and involvement of a third reviewer. Data extraction will be guided by the JBI template. Quantitative data will be reported descriptively, and a conventional content analysis will be undertaken for qualitative data.

**Inclusion criteria:**

This scoping review will include studies globally published in peer-reviewed academic journals in English involving PwMS that report on implementation strategies for live, professional-led psychological interventions. Pharmacological studies or studies focusing only on effectiveness of psychological interventions will be excluded.

## Introduction

Multiple sclerosis (MS) is a neurodegenerative condition that primarily affects the central nervous system [1], and the prevalence of MS has increased worldwide since 2013 [2]. People with MS (PwMS) report high levels of anxiety, depression, fatigue and stress due to disease unpredictability and an overall chronic disease burden [3,4]. While drug treatments may provide some relief, studies show that they may also be counterproductive due to their common side effects including increased fatigue, cognitive impairment and disability [5]. Psychological interventions (also known as psychological therapies) such as cognitive behavioural therapy, support groups and mindfulness-based interventions provide a safe and alternative treatment option for PwMS, and there is emerging evidence related to their efficacy and use in this population [6–8].

Previous systematic reviews have investigated the efficacy of various psychological therapies for PwMS [9–11]. The authors concluded that these types of interventions may improve physical (e.g., fatigue) and psychological (e.g., anxiety) symptoms, contributing to an improvement of overall quality of life for this population [9]. While previous research has outlined implementation considerations from different perspectives in the context of mindfulness-based interventions for PwMS [12,13], there remains a need to examine implementation strategies more broadly. In addition to evaluating the efficacy of psychological interventions for this population, it is also crucial to investigate how these interventions are implemented in practice to support their uptake and sustainability. This scoping review protocol outlines proposed steps to address this gap.

Implementation of healthcare interventions involves translating evidence-based interventions from research into routine clinical care [14]. More specifically, *implementation strategies* are methods or techniques used to enhance the adoption, implementation, and sustainability of a clinical program or practice [15]. A useful framework to categorize and assess implementation strategies is the Expert Recommendations for Implementing Change (ERIC) framework [15]. Strategies identified in ERIC highlight drivers for implementation success across multiple contexts. This scoping review protocol draws upon this framework as it provides useful guidance to identify key implementation strategies that influence the uptake and impact of an intervention.. Components such as patient engagement, training and clinical integration and infrastructure support [14] need to be explored to contextualize efficacy, assess impact and inform future programs to maximize benefits for PwMS.

Furthermore, one persistent gap in the field of implementation science is the lack of equity, diversity, inclusion and accessibility (EDIA) considerations when developing and implementing new healthcare interventions [16]. Incorporating EDIA considerations, particularly through an intersectional lens, can improve accessibility of healthcare interventions for marginalized and underserviced groups [16]. As Fahim and colleagues note, this approach recognizes how overlapping social identities (e.g., race, gender, socioeconomic status) and structural inequities shape individuals’ experiences and influence the implementation process [17]. Specifically, an understanding of these factors is required to investigate potential barriers and facilitators to implementation, associated implementation strategies, and sustainability of these programs [17] for all PwMS. This lens is particularly important as the rates of MS continue to rise globally (e.g., Iran, Western Europe etc) [2]. An investigation into the implementation and sustainability of psychological interventions will provide policymakers and healthcare professionals with a better understanding of the effectiveness, scalability and adoption of these services in practice. This scoping review protocol integrates considerations for investigating EDIA factors in this context, as it can provide important insight into implementation strategies that promote EDIA factors at the outset. These are crucial elements to ensure that these interventions are both accessible and focused on the needs of PwMS, which can ultimately maximize their mental health and overall wellbeing.

The objective of this paper is to standardize a scoping review protocol for completion of a scoping review. The aims of the proposed scoping review are to: 1) determine the extent of the literature on which implementation strategies have been used to develop, deliver, and sustain psychological interventions for PwMS and 2) investigate how EDIA considerations are embedded within these implementation strategies and psychological interventions more broadly.

## Methods

A scoping review was chosen to allow for a broad and comprehensive investigation of the existing literature. It will allow for the identification of both the sources and types of evidence available in this research area. A scoping review methodology will facilitate the inclusion of a variety of study designs (i.e., RCT, mixed methodology, observational, etc.). The research objectives do not focus on assessing intervention efficacy but will rather explore the evidence available to determine what has been done. A scoping review will provide an overview of the existing literature, map the existing implementation strategies that have been developed or used in the literature and highlight future areas of research in this context.

The Joanna Briggs Institute (JBI) scoping review methodology guidance [18] will be used to conduct this scoping review. The reporting of this protocol follows PRISMA-P [19] (S1 File), and The Preferred Reporting Items for Systematic Reviews and Meta-Analyses extension for Scoping Reviews (PRISMA-ScR) [20] will be used as a guideline for reporting our scoping review findings. A PRISMA-ScR flow diagram will provide a visual presentation and breakdown of the studies that were screened, excluded and included into the final review. The diagram will identify the number of articles retrieved from the relevant sources and reasons for exclusion at the full text stage.

### Protocol and registration

The protocol was registered on Open Science Framework on June 30, 2025. Registration DOI: https://doi.org/10.17605/OSF.IO/P2UCR.

### Inclusion criteria

#### Population.

The population of interest for this scoping review include PwMS. Psychological interventions must be outlined as specific to this population to be included in the final analysis.

#### Concept.

The concept being explored in this scoping review is implementation strategies for promoting the implementation of psychological interventions for PwMS. Studies will be included in the final review if they meet the following criteria: 1) primary original research of any study design, 2) full text available in English, 3) involves PwMS, diagnosed as such at the time of the study (inclusive of all types of MS such as relapsing remitting, primary progressive, etc.), 4) data from PwMS group is reported/can be extracted as a separate group (if multiple populations targeted), 5) involves a psychological intervention (defined as activities aimed at changing behaviors, feelings, and emotional states) [21]. Only interventions involving live interaction with a trained health professional will be included, as the American Psychological Association notes that psychotherapy comprises “any psychological service provided by a trained professional that primarily uses forms of communication and interaction” [22] and 6) addresses at least one implementation strategy based on the cluster of the ERIC strategies [23] (use of evaluative and iterative strategies; provide interactive assistance; adapt and tailor to context; develop knowledge user interrelationships; train and educate stakeholders; support clinicians; engage consumers; utilize financial strategies; and change infrastructure). Studies will be excluded if they do not meet an inclusion criterion and/or meet one of the following exclusion criteria: 1) interventions involve pharmacological or surgical interventions (unless combined with a psychological therapy), 2) data cannot be extracted from full text, 3) only assesses effectiveness of a psychological intervention (unless the implementation strategy is the intervention or it is a hybrid effectiveness-implementation study), 4) full text not available and 5) protocol paper or conference abstract.

#### Context.

Both qualitative and quantitative studies will be included. Articles will not be limited by publication year. For feasibility purposes, we will only consider studies published in English. Studies published in peer-reviewed academic journals from any geographical areas will be included if they meet the inclusion criteria.

#### Types of sources.

This review will consider all study designs that incorporate primary research. Included studies must directly describe implementation strategies from the ERIC taxonomy for psychological interventions for PwMS.

#### Search strategy.

An academic research librarian will support the development of an electronic database search strategy. An initial search will be conducted in Medline (Ovid) (S2 File) with MeSH terms and text words for the following three concepts: multiple sclerosis, psychological therapies, and implementation science. The review team will identify additional search terms and synonyms to include. The initial Medline search will be peer-reviewed using the Peer Review of Electronic Search Strategies (PRESS) guidelines [24]. The final search strategy will be translated across the following databases: CINAHL (EBSCOhost), Embase (Ovid) and PsychInfo (Scopus). The search will not be limited by the publication year. Reference lists across included studies will be screened for identifying additional studies.

### Study/source of evidence selection

Citations identified from the search will be uploaded into the software Covidence, which will remove duplicates. Two reviewers will screen a random sample of 100 titles and abstracts to establish inter-rater reliability with a Cohen’s Kappa of 0.80 indicating substantial agreement [25]. Once inter-rater reliability has been established, titles and abstracts will be screened by two or more reviewers based on the inclusion criteria. Each reviewer can select one of the following functions for each title/abstract: ‘include’ and ‘exclude’. The reviewers will discuss interpretations of operational definitions to ensure clarity, understanding and consistency when screening for studies to include. Studies where a reviewer is unsure whether it meets the inclusion criteria will be included in the next full text review phase. Full texts of studies identified as relevant in the titles and abstracts stage will be retrieved in Covidence, which will be screened by two or more reviewers. Both reviewers will independently screen studies in Covidence across the same criteria for inclusion into the final scoping review. The reasons for exclusion will be documented and reported in the scoping review. Disagreements about inclusion and exclusion of studies at each stage of the review process will be resolved through discussion and a third reviewer, if necessary.

### Data extraction

Data from included full-texts will be systematically charted independently by two reviewers in Covidence using a data extraction template guided by the JBI template and the review team. The following information will be extracted and documented: author(s), year of publication and country of publication, objective/purpose of study, study design (e.g., RCT, retrospective etc.), type of psychological intervention (e.g., cognitive behavioural therapy, mindfulness etc.), delivery format (e.g., pre-recorded workshops, live sessions etc.), description of implementation strategy guided by the nine ERIC clusters, other implementation considerations not directly captured by the ERIC clusters (including frameworks used as guidance), primary outcomes, EDIA considerations guided by the PROGRESS -Plus framework [26] (e.g., age, race, socioeconomic status etc.) and key recommendations.

Data extraction based on the aforementioned characteristics will be piloted in duplicate for the first five included studies to ensure consistency, interpretation and validity across reviewers. The template will be modified as necessary based on this exercise. Once the data extraction template is confirmed through this process, the rest of the included studies will be extracted for data in duplicate by two reviewers. Reviewers will meet to discuss and resolve any discrepancies or inconsistencies once extraction is complete. If required, a third reviewer will be asked to adjudicate/seek consensus. The review team will contact authors of studies where there is missing, or additional data required.

### Data analysis

The results will be collated, summarized and reported by the primary reviewer (AS). Quantitative findings will be organized into appropriate tables, summarized using descriptive statistics (e.g., means, frequencies/percentages) and a narrative synthesis as appropriate. A conventional content analysis [27] (presented as descriptive synthesis) will be used for included qualitative studies.

### Status and timeline

An initial search has been translated across the four databases with a preliminary assessment of titles and abstracts. Record screening is anticipated to be complete by the end of November 2025. Data extraction is expected to be complete by the end of December 2025. Results are expected to be synthesized by the end of January 2026.

## Discussion

This scoping review will provide novel insights into strategies used to implement psychological interventions for PwMS. These strategies can be used to optimize the implementation of psychological interventions in the future and in diverse contexts as well as inform the development of a toolkit of implementation strategies to optimize implementation. The identification of implementation strategies used across these interventions can further highlight important gaps in current implementation practices such as lack of standardization and underutilized strategies. This review further seeks to identify EDIA considerations embedded within psychological intervention development and implementation strategies to inform future programs to continuously evaluate implications for marginalized and underserviced groups. It will also highlight implementation strategies that integrate and promote EDIA considerations.

Findings from this scoping review will be disseminated through traditional knowledge mobilization channels such as conferences (e.g., endMS conference) and publication in a peer-reviewed academic journal (e.g., Disability and Rehabilitation Journal). Key knowledge users such as policy makers, clinicians and groups who are involved with building psychological interventions can use findings from the review to maximize the development, and implementation of future interventions with considerations related to EDIA characteristics.

This review will have several strengths. The search strategy will be developed with an academic research librarian and peer reviewed using PRESS [19], ensuring a rigorous approach to identifying relevant studies. The use of comprehensive frameworks such as ERIC and PROGRESS-Plus will systematically categorize implementation and EDIA characteristics, contextualized within existing literature and highlighting critical gaps. The review team will include individuals with clinical and academic backgrounds with expertise in scoping reviews and implementation science, ensuring relevancy and applicability to diverse contexts.

Some limitations of this scoping review are anticipated. First, implementation science includes a broad scope of evidence and conceptualizations vary in healthcare literature. The inherent heterogeneity of the term ‘implementation’ makes identifying relevant studies challenging. For example, the term ‘implementation’ may not be included in studies, even though aspects of implementation such as training and staffing considerations were included. To mitigate this, the search strategy included broad terms related to implementation science based on previous literature in the field. Due to feasibility constraints, this review will not include a scope of the grey literature, which may result in the exclusion of implementation literature outside of peer-reviewed contexts.

## Supporting information

S1 File PRISMA-P 2015 (Preferred reporting items for systematic review and meta-analysis protocols) checklist. (DOCX)

S2 File MEDLINE search strategy draft. (DOCX)
