# Supplementary material for: The use of implementation strategies to promote the uptake of psychological therapies among people with multiple sclerosis: A scoping review protocol
Source: PLoS One. 2025 Nov 20;20(11):e0337105. doi: 10.1371/journal.pone.0337105 (PMC12633889; doi:10.1371/journal.pone.0337105)
Supplement: S2 File — (DOCX) [file pone.0337105.s002.docx]

## Medline

**Database:** Ovid MEDLINE(R) ALL <1946 to February 11, 2025>

| **#** | **Query** | **Results from 10 Feb 2025** |
| --- | --- | --- |
| 1 | exp Multiple Sclerosis/ | 74,481 |
| 2 | Myelitis/ or Myelitis, Transverse/ | 4,789 |
| 3 | Optic Neuritis/ or Neuromyelitis Optica/ | 11,244 |
| 4 | Encephalomyelitis, Acute Disseminated/ | 2,193 |
| 5 | Demyelinating Autoimmune Diseases, CNS/ | 581 |
| 6 | ((multiple or disseminat* or multiplex or insular) adj sclerosis).ti,ab,kf,jw. | 99,888 |
| 7 | ((clinically or radiologically) adj isolated syndrome).ti,ab,kf,jw. | 1,971 |
| 8 | (myelit* or neuromyelit* or devic or neuropticomyelit* or myelooptic* or myelo optic* or myeloptic*).ti,ab,kf,jw. | 12,275 |
| 9 | (acute disseminat* encephalomyelit* or acute demyelinat* encephalomyelit*).ti,ab,kf,jw. | 2,540 |
| 10 | (demyelin* adj3 disease*).ti,ab,kf,jw. | 10,464 |
| 11 | (optic* neuritis or neuritis optic*).ti,ab,kf,jw. | 8,069 |
| 12 | (ADEM or NMOSD or PwMS).ti,ab,kf,jw. | 6,796 |
| 13 | or/1-12 [Multiple Sclerosis] | 129,538 |
| 14 | exp Psychotherapy/ | 229,408 |
| 15 | Psychology, Applied/ or exp Counseling/ | 51,133 |
| 16 | Psychological Techniques/ or Interview, Psychological/ | 15,943 |
| 17 | Psychoanalysis/ or exp Sensory Art Therapies/ | 67,758 |
| 18 | (psychotherap* or psycho-therap*).ti,ab,kf,jw. | 73,792 |
| 19 | (group therap* or (group* adj2 technique*)).ti,ab,kf. | 13,501 |
| 20 | ((inter-personal or interpersonal) adj2 (psychotherap* or therap* or technique*)).ti,ab,kf. | 1,921 |
| 21 | counsel?ing.ti,ab,kf. | 130,745 |
| 22 | supportive therap*.ti,ab,kf. | 6,069 |
| 23 | (cognitive adj2 behavio?r* adj3 (therap* or technique* or intervention* or train* or treatment* or psychotherap* or program* or method* or approach*)).ti,ab,kf. | 34,417 |
| 24 | CBT?.ti,ab,kf. | 17,744 |
| 25 | (acceptance adj2 commitment adj3 (therap* or technique* or intervention* or train* or treatment* or psychotherap* or program* or method* or approach*)).ti,ab,kf. | 2,282 |
| 26 | (behavio?r* adj2 (therap* or technique* or treatment* or modification?)).ti,ab,kf. | 60,431 |
| 27 | (conditioning adj2 therap*).ti,ab,kf. | 748 |
| 28 | ((animal? or pet? or equine?) adj2 therap*).ti,ab,kf. | 4,098 |
| 29 | hippotherap*.ti,ab,kf. | 265 |
| 30 | mindful*.ti,ab,kf. | 20,306 |
| 31 | ((art or arts or artistic* or artist* or play or danc*) adj2 therap*).ti,ab,kf. | 23,890 |
| 32 | (psychodynamic adj (psychotherap* or therap* or technique* or analys#s)).ti,ab,kf. | 1,967 |
| 33 | (psychoanalytic* or psycho-analytic* or psychoanalys#s or psych-oanalys#s).ti,ab,kf. | 18,660 |
| 34 | (psychologic* adj3 (therap* or technique* or intervention* or train* or treatment* or program* or method* or approach* or analys#s)).ti,ab,kf. | 36,326 |
| 35 | anger management.ti,ab,kf. | 497 |
| 36 | ((aversion or aversive) adj2 therap*).ti,ab,kf. | 261 |
| 37 | neurofeedback?.ti,ab,kf. | 2,611 |
| 38 | (feedback? adj2 (eeg? or brainwave? or alpha or electromyograph* or electroencephalograph* or biofeedback?)).ti,ab,kf. | 477 |
| 39 | (cognitive adj2 (restructur* or refram* or re-fram* or re-structur* or reapprais* or re-apprais* or revaluat* or re-evaluat*)).ti,ab,kf. | 3,135 |
| 40 | (cognitive adj2 remediation*).ti,ab,kf. | 1,682 |
| 41 | desensitization.ti,ab,kf. | 25,653 |
| 42 | ((exposure? or flooding? or implosive or immersion) adj3 (therap* or technique* or psychotherap*)).ti,ab,kf. | 10,892 |
| 43 | (eye movement* adj3 (desensiti* or reprocess* or process*)).ti,ab,kf. | 1,383 |
| 44 | EMDR.ti,ab,kf. | 985 |
| 45 | (relax* adj2 therap*).ti,ab,kf. | 2,915 |
| 46 | meditat*.ti,ab,kf. | 9,584 |
| 47 | ((crisis or brief) adj2 (intervention? or advice or treatment)).ti,ab,kf. | 13,580 |
| 48 | (emotion* adj2 focused therap*).ti,ab,kf. | 260 |
| 49 | process-experiential therap*.ti,ab,kf. | 3 |
| 50 | (gestalt adj2 therap*).ti,ab,kf. | 111 |
| 51 | (hypnosis or hypnotist or hypnotism or hypnoanalysis or hypnogenesis or hypnotherap* or mesmerism or hypnotic*).ti,ab,kf. | 26,028 |
| 52 | autogenic training.ti,ab,kf. | 644 |
| 53 | (progressive adj2 relaxation?).ti,ab,kf. | 1,209 |
| 54 | (suggestion? or autosuggestion?).ti,ab,kf. | 99,773 |
| 55 | ((guided or psychotherap*) adj2 imagery).ti,ab,kf. | 1,075 |
| 56 | (logotherap* or logo-therap*).ti,ab,kf. | 179 |
| 57 | ((mentalis* or mentaliz*) adj3 (therap* or psychotherap* or treatment? or technique?)).ti,ab,kf. | 473 |
| 58 | (narrative? adj2 therap*).ti,ab,kf. | 747 |
| 59 | ((person or client) adj center* adj2 (therap* or psychotherap*)).ti,ab,kf. | 240 |
| 60 | ((nondirective or non-directive or rogerian) adj2 therap*).ti,ab,kf. | 138 |
| 61 | free association?.ti,ab,kf. | 690 |
| 62 | transactional analys#s.ti,ab,kf. | 201 |
| 63 | (psychosocial adj3 (therap* or technique* or intervention* or train* or treatment* or program* or method* or approach* or analys#s)).ti,ab,kf. | 19,127 |
| 64 | ((psychotherapeutic or "psychiatric therapeutic") adj2 process*).ti,ab,kf. | 467 |
| 65 | (abreaction? or cathars#s or transference? or countertransference?).ti,ab,kf. | 8,946 |
| 66 | therapeutic alliance?.ti,ab,kf. | 3,979 |
| 67 | (motivat* adj2 interview*).ti,ab,kf. | 6,491 |
| 68 | (pastoral adj2 (care or psycholog*)).ti,ab,kf. | 953 |
| 69 | ((couple or couples or marriage or marital or family) adj2 therap*).ti,ab,kf. | 7,153 |
| 70 | (drama therap* or psychodrama?).ti,ab,kf. | 514 |
| 71 | (role play* or roleplay*).ti,ab,kf. | 26,164 |
| 72 | or/14-71 [Psychological Therapies] | 783,430 |
| 73 | 13 and 72 | 2,777 |
| 74 | Implementation Science/ | 1,671 |
| 75 | Translational Science, Biomedical/ or Translational Research, Biomedical/ or "Diffusion of Innovation"/ or Information Dissemination/ | 51,220 |
| 76 | Capacity Building/ or Professional Practice Gaps/ or Evidence Gaps/ or exp Organizational Innovation/ or Organizational Objectives/ | 51,519 |
| 77 | Knowledge/ or Knowledge Discovery/ or Knowledge Management/ | 16,039 |
| 78 | (implement* or adopt* or apply or application* or disseminat* or diffus* or transl* or transfer* or uptak* or scale? or scaling or scale?-up).ti,ab,kf. | 6,305,128 |
| 79 | ((knowledge or information or expertise or research or evidence or findings or resource* or experience* or perspective*) adj3 (shar* or mobili* or exchang* or broker* or product* or creat* or manag* or acqui* or accru*)).ti,ab,kf. | 241,778 |
| 80 | (know-do or research-to-practice or research-to-action or knowledge-to-practice or knowledge-to-action or evidence-to-practice or evidence-to-action).ti,ab,kf. | 42,176 |
| 81 | (re-aim or cfir).ti,ab,kf. | 3,105 |
| 82 | ((profession* or interprofession* or interdisciplin* or multiprofession* or multidisciplin* or disciplin* or organi#ation* or institution* or continuing or social* or group* or peer*) adj3 knowledge).ti,ab,kf. | 19,927 |
| 83 | ((capacit* or skill* or competenc* or expertise or knowledge) adj3 (build* or develop* or improv* or enhanc* or upgrad* or gap or gaps)).ti,ab,kf. | 228,143 |
| 84 | (step? or goal? or outlin* or strateg* or barrier* or facilitator* or innovat*).ti,ab,kf. | 3,594,852 |
| 85 | or/74-84 [Implementation (with some knowledge translation terms)] | 9,027,780 |
| **86** | **73 and 85** | **1,190** |
